# From Tpex to Tex: a journey through CD8^+^ T cell responses in cancer immunotherapy

**DOI:** 10.1038/s41392-023-01595-1

**Published:** 2023-09-04

**Authors:** Julia M. Messmer, Maike Effern, Michael Hölzel

**Affiliations:** Institute of Experimental Oncology, University Hospital Bonn, University of Bonn, 53127 Bonn, Germany

**Keywords:** Tumour immunology, Tumour immunology

In a recent study published in *Cell*, Rahim et al. show that a subset of progenitor exhausted CD8^+^ T cells (Tpex) in uninvolved, regional lymph nodes (uiLNs) are clonally related to exhausted CD8^+^ T cells (Tex) in tumors of patients with head and neck squamous cell carcinoma (HNSCC) and following adjuvant immune checkpoint blockade (ICB) these clones expanded in the tumor suggestive of migration from the uiLNs and differentiation into an exhaustion state.^1^ The work by Rahim et al. underscores the great value of neoadjuvant ICB clinical trials to better understand the dynamics of T cell responses in patients.

Rahim et al. comprehensively profiled immune cells in the tumor microenvironment (TME) and in matched uiLNs and/or metastatic lymph nodes (metLNs) of patients with metastatic HNSCC using mass cytometry. Tpex were found at a higher frequency in the uiLNs compared to matched tumor tissue, while Tex and effector memory CD8^+^ T cells were enriched in the tumor. Combining single-cell RNA sequencing with TCR-seq and/or single-cell protein expression to assess matched LN and tumor tissues from HNSCC patients revealed CD8^+^ T cell clonality between the TME and the LNs. Shared T cell clones in the uiLNs belonged to the Tpex phenotype, whereas their counterparts in the TME were more likely to exhibit an intermediate exhausted CD8^+^ T cells (Tex-int)/terminally exhausted CD8^+^ T cells (Tex-term) phenotype. Multiplexed ion beam imaging revealed that in uiLNs from patients treated with surgery only, Tpex and Tex-int were found in cellular neighborhoods with CD4^+^ T cells, regulatory T cells (Tregs), CD4^+^ CD8^+^ T cells and dendritic cells (DCs). Pre-clinical mouse studies have shown that cDC1s (type I conventional dendritic cells) in tumor-draining LNs (tdLNs) maintain a reservoir of proliferative tumor-reactive Tpex and that boosting cDC1s increases Tpex cell frequencies and decreases tumor burden.^2^ Rahim et al. show that neoadjuvant ICB decreased frequencies of Tpex cells and increased frequencies of Tex-int in uiLNs and altered the spatial organization of the uiLNs increasing the numbers of neighboring DCs of both Tpex and Tex-int cells. Moreover, Tpex localized in closer proximity to Tex-int cells indicating activation and differentiation toward a Tex-int phenotype.

Expansion of activated CD8^+^ T cells in the blood has been associated with clinical responses to ICB in various tumor entities. After ICB treatment, Tpex cells were detected in the blood and in the primary tumor tissue only at low levels, implying that the overall decreased frequency of Tpex in treated uiLN is not caused by cell egress from the LNs. Instead, circulating Tex-int and terminally differentiated CD8^+^ effector memory cells with enhanced proliferation were detected in the blood and significantly increased after neoadjuvant ICB. This is in line with other literature describing CD8^+^ T cells from tdLNs as developmental precursors of the Tex cells in tumors maintained by continuous migration.^3^ By protecting T cells from terminally differentiating, the tdLNs have a critical function in maintaining anti-tumor CD8^+^ T cell immunity.

This paper highlights that uiLNs contribute to a successful anti-tumor immune response as they harbor a pool of stem-like CD8^+^ T cells giving rise to T cells involved in the anti-tumor immunity. This represents an opportunity to critically discuss the clinical practice of LN dissection, in particular in the context of ICB-based regimens. LNs are frequently dissected during tumor surgery as LN involvement is an important prognostic factor and an essential element for disease staging. However, for some cancer entities such as melanoma, breast or gynecologic cancers, LN dissection often provides little to no therapeutic benefit. It is beyond the scope of this publication but it raises the question whether it is always beneficial to remove non-metastatic LNs.

Rahim et al. also addressed the impact of metastasis on LN functionality. They compared CD8^+^ T cell phenotype and spatial organization of uiLNs and metLNs from non-treated and ICB-treated patients. Tpex cells were almost non-existent in metLNs, whereas Tex-term were more abundant. Moreover, Tpex and Tex-int reside in an immunosuppressive cellular niche in metLNs, characterized by the abundance of proliferative Tregs and CD4^+^ T cells with a more naïve or quiescent phenotype, and DCs expressing immune regulatory molecules. Patients with metLNs had a more blunted response to neoadjuvant ICB. Other literature has shown that LN metastases are epigenetically rewired harboring a tumor intrinsic interferon response program that promotes NK cell evasion and LN colonization. LN metastases induce antigen-specific Tregs and resist T cell-mediated cytotoxicity resulting in metastasis-promoting tumor-specific immune tolerance.^4^

It still remains unclear whether lymph node dysfunction is the cause or consequence of metastatic tumor spread and increased disease burden. These questions could be addressed by refined pre-clinical tumor models implementing spatial and longitudinal analysis of anti-tumor immunity. Improved pre-clinical models ideally show slower growth and spontaneous metastasis to better recapitulate human disease. However, the majority of mouse studies rely on fast-growing transplantable syngeneic models and outgrowth of the primary tumor is often a limiting factor. One solution could be surgical removal of the primary lesion allowing the investigation of an ongoing immune response and still offering an opportunity for clinical intervention. Moreover, extensive analyses of various murine and patient LNs would allow an in-depth characterization of the heterogeneity of LN architecture. It has been shown that LN dissection in mice reduces immunotherapy efficacy suggesting that tumor-antigen-specific immunity is not just limited to the tdLN but may extend to other secondary lymphoid organs.^5^ This is however difficult to model in mouse models.

**Fig. 1:**
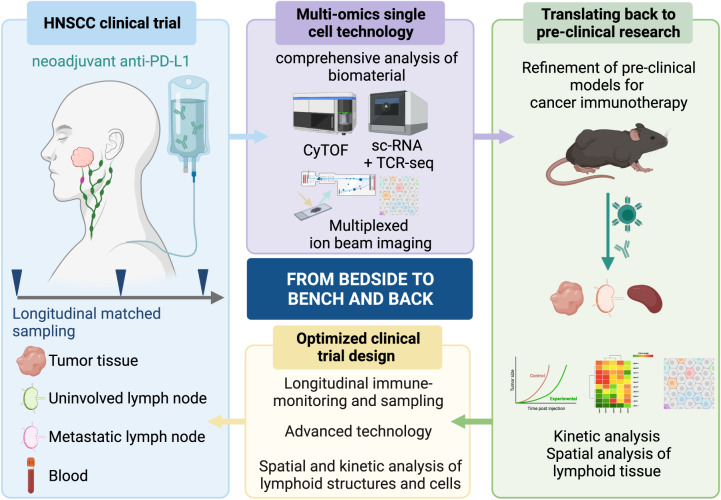
Neoadjuvant cancer immunotherapy trials and translational companion studies like Rahim et al. provide unique opportunities to guide the development and benchmarking of pre-clinical models to reiterate knowledge and improve the clinical trial design. Figure created with BioRender.com

Despite the limited number of patients showcased, the data in this paper describes novel insights into CD8^+^ T cell dynamics and cellular interactions in the context of human cancer, which is well supported by various pre-clinical studies. The publication illustrates how valuable a good clinical trial design allowing the collection of longitudinal matched tissue samples can be for understanding human cancer in greater detail. With the advent of multiplex approaches, tissues from LN dissections could help guide future treatment strategies by evaluating the T cell repertoires and cellular interactions. It is an opportunity to investigate to which extent immunity extends beyond the tdLN. In summary, we propose that neoadjuvant ICB clinical trials and translational studies like Rahim et al. are instrumental to guide the development of improved pre-clinical models that in turn allow the exploration of novel therapeutic concepts and refine the clinical trial design (Fig. 1).
